# Extracellular gamma-synuclein promotes tumor cell motility by activating β1 integrin-focal adhesion kinase signaling pathway and increasing matrix metalloproteinase-24, -2 protein secretion

**DOI:** 10.1186/s13046-018-0783-6

**Published:** 2018-06-15

**Authors:** Caiyun Liu, Like Qu, Chuanke Zhao, Chengchao Shou

**Affiliations:** 1 Key Laboratory of Carcinogenesis and Translational Research (Ministry of Education/Beijing), Beijing, China; 2 Department of Biochemistry & Molecular Biology, Peking University Cancer Hospital & Institute, Beijing, China

**Keywords:** Gamma-synuclein, β1 integrin, Focal adhesion kinase, MMP-2, Motility, Activation

## Abstract

**Background:**

Increasing evidence reveals a significant correlation between gamma-synuclein (SNCG) level and tumor invasion and metastasis in various human cancers. Our previous investigation showed that SNCG could secrete into extracellular environment and promoted tumor cell motility, but the mechanism is unknown.

**Methods:**

The membrane binding ability of SNCG was characterized by immunohistochemical staining, immunofluorescence staining and fractionation of colorectal cancer (CRC) cell membrane. Association between SNCG and β1 integrin was validated by coimmunoprecipitation and far Western blot. After inhibition of β1 integrin and focal adhesion kinase (FAK), effect of SNCG on cell motility was measured by transwell chamber assays and changes of protein levels were detected by Western blot. Association between SNCG and activated β1 integrin levels in human CRC tissues was determined by Spearman’s rank correlation analysis. Secreted proteins in conditioned medium (CM) were screened by antibody array.

**Results:**

Extracellular SNCG bound β1 integrin on CRC cell membrane and increased levels of activated β1 integrin and FAK. Correspondingly, SNCG-enhanced cell motility was counteracted by knockdown or inhibition of β1 integrin or FAK. Further study revealed that high SNCG level indicated poor outcome and SNCG levels positively correlated with those of activated β1 integrin and phospho-FAK (Tyr^397^) in human CRC tissues. Additionally, extracellular SNCG promoted secretion of fibronectin (FN), vitronectin (VN), matrix metalloproteinase (MMP)-2, and MMP-24 from HCT116 cells. Protease activity of MMP-2 in the CM of HCT116 cells was increased by treatment with SNCG, which was abolished by inhibiting β1 integrin.

**Conclusion:**

Our results highlight the potential role of SNCG in remodeling extracellular microenvironment and inducing β1 integrin-FAK signal pathway of CRC cells.

**Electronic supplementary material:**

The online version of this article (10.1186/s13046-018-0783-6) contains supplementary material, which is available to authorized users.

## Background

Gamma-Synuclein (SNCG), the third member of the neuronal protein synuclein family, participates in the pathogenesis of several types of cancer and some neurodegenerative diseases. It has been shown that SNCG promotes tumor cells migration and invasion in *in vitro* assays [1–3] as well as metastasis in animal models [1]. Furthermore, elevated SNCG levels in primary tumors positively correlated with distant metastasis or tumor recurrence in patients of breast cancers [4], colon cancer [5, 6], and pancreatic cancer [7], and associated with poor prognosis in a number of human cancers of different origins [5–8]. SNCG is a soluble protein predominantly distributed in the cytosol of tumor cells and functions both intra- and extra-cellularly [3], just like alpha-synuclein (SNCA), another member of synuclein family [9]. Inside cells, SNCG is implicated in regulating microtubule [10], stimulating membrane-initiated estrogen signaling [11], protecting Akt and mTOR and rendering tumor resistance to Hsp90 disruption [12], interacting and regulating insulin-like growth factor I (IGF-I) receptor expression [13], and inhibiting stress- and chemotherapy drug-induced apoptosis [14]. As SNCG lacks a signal sequence that could direct it across the classical endoplasmic reticulum-Golgi secretory pathway, secretion of SNCG occurs via a non-classical secretory pathway [3]. Increased SNCG levels were found in tumor cell culture medium [15], serum [16] and urine [17, 18] from various cancer patients.

Overexpression of SNCG in colon adenocarcinoma LS 174T cells led to increased adhesion to collagen and fibronectin [2]. Integrin, the major fibronectin receptor, has been linked to both tumor suppression and progression in different human malignancies [19]. β1 integrin is involved in gastric cancer progression [20, 21], promotes tumor cell migration and invasion [21–23], regulates invadopodia formation [23], mediates resistance to adjuvant and ionizing radiation therapy [22, 24–26], and plays a key role in regulating the switch of cancer cells from a dormant state to active proliferation and metastasis [26]. β1 integrin receptor binds extracellular matrix (ECM) to regulate multiple signaling events such as FAK/AKT or FAK/ERK pathway [20, 25, 27] and significantly correlates with patient outcome and may be a potential prognostic biomarker in TNBC patient survival [22]. These studies reminded us to unveil whether β1 integrin could have functional or/and physical association with SNCG in tumorigenesis.

Recognition of matrix molecules by cell surface integrins and the subsequent degradation of the matrix are important mechanisms in cell invasion. Integrins are the regulators of the expression of matrix metalloproteinases (MMPs), secretion and activation of the latent protease at the cell surface [28]. MMP-2 and -9 have been recognized as major contributors to the proteolytic degradation of ECM during tumor invasion and their elevated expression is positively correlated with tumor progression, metastasis, and poor overall prognosis [29, 30]. SNCG levels positively correlated with those of MMP-9 in breast cancer tissues [31] and SNCG overexpression in retinoblastoma cells upregulated the expression of MMP9 through activation of AP-1 cis element [32]. Based on our previous results and studies mentioned above, the purpose of this study was to reveal the mechanism by which extracellular SNCG promoted tumor cell motility. In the current study, we demonstrated that extracellular SNCG bound β1 integrin and promoted migration and invasion of CRC cells by β1 integrin activation, FAK phosphorylation, and secretion of MMP-24 and MMP-2. Furthermore, positive correlations among SNCG, activated β1 integrin, and phospho-FAK (Y^397^) were revealed in human CRC tissues.

## Methods

### Cell lines and reagents

Human CRC cell lines HT29, HCT116, DLD-1, RKO, CL187, LS 174T, SW480, and LOVO, were obtained from the American Type Culture Collection and cultured in RPMI-1640 (GBICO) with 10% fetal bovine serum (FBS) at 37°C under 5% CO_2_ in air.

Costar 3422 Transwell plates (6.5 mm Insert, 24 well Plate, 8.0 μm) were from Corning Incorporation. Matrigel was purchased from BD Biosciences (San Diego, CA, USA). RGD peptide (GRGDNP) (sc-201176), FAK inhibitor 14 (sc-203950), and MMP-2 Inhibitor I (sc-204092) were purchased from Santa Cruz (Santa Cruz, CA, USA).

Mouse anti-human β1 integrin monoclonal antibody (mAb) (specifically recognizing the active conformation) is from BD Biosciences. Detailed information about other antibodies was listed in Additional file 1: Table S1. Mouse anti-SNCG mAb was generated and characterized as in Reference [15]. HRP-conjugated goat anti-rabbit/mouse immunoglobulin (IgG), and FITC-conjugated goat anti-mouse IgG were from Santa Cruz. Recombinant GST, GST-SNCG, and untagged SNCG were expressed and purified as previously reported [3, 15].

### Specimens

The 250 archival paraffin-embedded colon cancer specimens were obtained from the Department of Pathology, Peking University Cancer Hospital & Institute (Beijing, China). All patient details and exclusion criteria have been described previously [5]. Specimens from patients were diagnosed histopathologically and staged according to the TNM-International Union against Cancer classification system. The clinicopathologic characteristics of patients were described in Additional file 1: Table S2. The 37 frozen colon cancer tissues samples used in Western blot analysis for SNCG, β1 integrin, and p-FAK levels were randomly collected from the cohort of 250 cases. All of patients involved in this study consented to participate in the study and publication of its results. The study was approved and supervised by the Medical Ethic Committee of Peking University Cancer Hospital & Institute.

### Immunohistochemistry and evaluation of Immunohistochemical staining

Immunohistochemistry was performed and the immunohistochemical staining was evaluated as previously described [5].

### Immunocytofluorescence

Cells were seeded on coverslips and incubated in complete cell culture medium. After 16 h, cells were treated with 1 μmol/L of GST, GST-SNCG, BSA, or SNCG for 30-60 min, washed, fixed with ice methanol or 4% paraformaldehyde for 10 min. The slides were incubated with anti-SNCG overnight at 4°C, washed and incubated with FITC- or TRITC-conjugated secondary antibody. F-actin was visualized by staining with FITC-phallodin. Stained cell were analyzed using the Leica SP5 confocal system (Leica) with the x 60 oil-immersion objective.

### Analysis of cell membrane binding form of SNCG

**Fig. 1:**
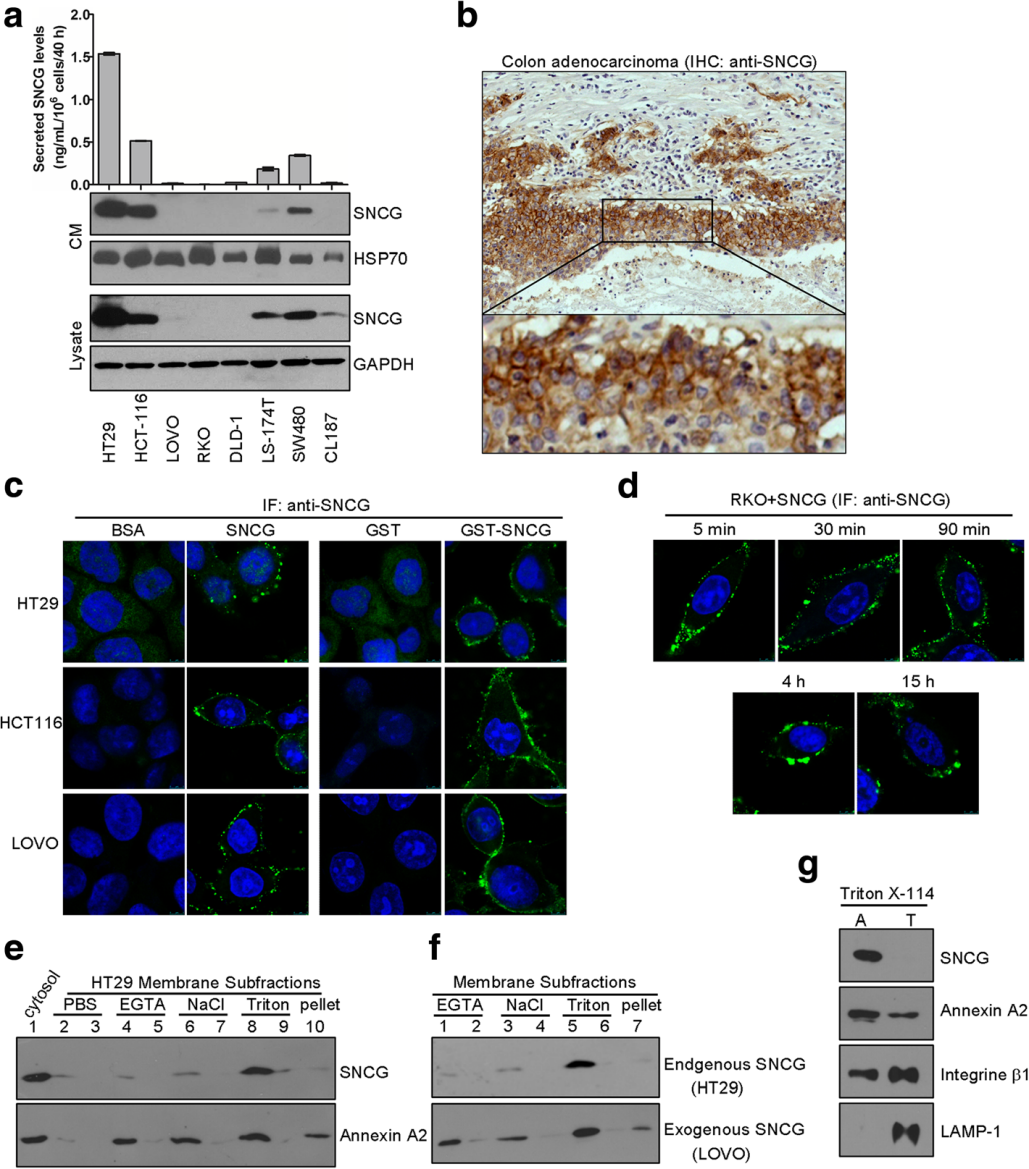
SNCG binds cell membrane and the binding form of SNCG on tumor cells was evaluated. **a**, Secreted SNCG levels positively correlate with intracellular SNCG levels in colorectal cancer (CRC) cell lines. SNCG levels in the conditioned medium (CM) were directly detected by the Sandwich ELISA (upper panel), and the corresponding concentrated CM and cell lysates were analyzed by Western blot (lower panel). HSP70 and GAPDH were respectively used as loading controls for CM and cell lysates. Representative results from three independent experiments were presented. **b**, Representative immunohistochemical staining for membrane SNCG (brown) with haematoxylin counterstain in human colon adenocarcinoma tissues. **c**-**d**, Immunofluorescent staining was performed as described under “Methods”. Both recombinant GST-SNCG and SNCG bound cell membranes in different CRC cells (**c**); Membrane-binding of exogenous SNCG in RKO cells was detected as early as five minutes after treatment and maintained at almost similar levels (**d**). The results shown are representative of at least six different fields observed in each experiment and of three similar independent experiments. **e**, Membrane binding form of SNCG was evaluated in HT29 cells. Cells were lysed and subfractionated as described in the Methods section. The cytosolic supernatant (lane 1), membrane subfractions (lanes 2-10) obtained by sequential washing twice with PBS (lanes 2-3), 5mM EGTA (lanes 4-5), 2 M NaCl (lanes 6-7), 1% Triton X-100 (lanes 8-9), and Triton X-100-resistant pellet (lane 10) were evaluated by Western blot using antibody against SNCG. Membrane protein Annexin A2 was used as a control. **f**, Comparison of cell membrane binding form of endogenous SNCG with exogenously added SNCG. Membrane subfractions from HT29 cells expressing endogenous SNCG (upper panel) and LOVO cells treated with exogenous SNCG protein (lower panel) were evaluated by specific antibody to SNCG. Membrane subfractions were successively washed twice with 5 mM EGTA (lanes 1-2), 2 M NaCl (lanes 3-4) and 1% Triton X-100 (lanes 5-6). Lane 7, Triton X-100-resistant pellet. **g**, Phase separation of hydrophilic and hydrophobic proteins. Membrane fraction was described under “Methods”. Aliquots of the aqueous (A) and detergent (T) phases were analyzed by Western blot analysis

Cell membrane fraction was obtained as described previously [3, 33]. Briefly, HT29 cells were collected in PBS buffer containing 1 mM EDTA and protease inhibitor cocktail, and disrupted by several rounds of freezing and thawing. After centrifuging for 20 min at 730 g, the supernatant was collected and centrifuged at 100, 000 g for 1 h at 4°C. Extraction of membrane binding proteins was collected as previously described [34], the membrane fraction was successively washed twice with PBS (Fig. 1e lane 2-3 and 1e lane 1-2), 5 mM EGTA (Fig. 1e lane 4-5 and 1f lane 3-4), 2 M NaCl (Fig. 1e lane 6-7 and 1f lane 3-4), 1% Triton X-100 (Fig. 1e lane 8-9 and 1f lane 5-6), respectively. All of the elutes (Fig. 1e, lane 2-9 and 1f lane 1-6), the cytosol (Fig. 1e lane 1) and Triton X-100-resistant pellet (Fig. 1e, lane 10 and 1f, lane 7) were evaluated by Western blot using anti-SNCG mAb and Annexin A2 was used as the control.

### Phase separation of EGTA-resistant protein in Triton X-114

This was done according to the method of Trotter [34]. Shortly, the EGTA-washed membrane fraction was resuspended in 200 μl of 10 mM Tris-HC1, pH 7.4, 150 mM NaCl, 1% TritonX-114 and incubated on ice for 5 min. The phase separation was induced by incubation at 30°C for 10 min. After centrifugation at 300 g for 10 min, the detergent phase and aqueous phase were analyzed for the presence of SNCG by Western blot.

### Small interfering RNA (siRNA) transfection

SiRNAs were transiently transferred with siRNA-Mate reagent (GenePharma, Suzhou, China) according to the manufacturer’s protocol. The target siRNA sequences against β1 integrin, FAK, negative control, and GAPDH were listed in Additional file 1: Table S3. The control siRNA sequence did not match any known human cDNA.

### Sodium Dodecyl Sulfate–Polyacrylamide Gel Electrophoresis (SDS-PAGE) and Western blot analysis

Based on the results of siRNA validation, cells were transfected with indicated siRNAs for 72 h, and then treated with GST or GST-SNCG (0.1 μmol/L) for 30 min. Alternatively, to confirm the effect of SNCG on β1 integrin and FAK signal pathway, HCT116 cells were treated with integrin inhibitor RGD (Arg-Gly-Asp) peptides (200 μmol/L) or FAK inhibitor 14 (50 μmol/L) for 4 h, then GST or GST-SNCG (0.1 μmol/L) were added for 30 min. Cell were washed with PBS twice and harvested with RIPA buffer containing 1 mM EDTA, 100 μM sodium orthovanadate, 100 μM sodium fluoride, 100 μM phenylmethanesulfomyl fluoride (PMSF), and protease inhibitor cocktail. The protein concentration was quantified by BCA assay. Protein lysates (30 μg) were resolved by SDS-PAGE, transferred to a nitrocellulose membrane (GE Healthcare, Pittsburgh, PA, USA) and probed with specific primary antibodies.

### Far-Western blot

Cell lysates were separated by native PAGE and then transferred to a nitrocellulose membrane. The membrane was blocked and probed with 10 ng of purified SNCG (bait protein) overnight at room temperature. HRP-labeled anti-SNCG mAb was used to detect β1 integrin (prey protein) on the membrane.

### Migration and invasion assays

HCT116 or SW480 cells were transfected with indicated siRNAs for 48 h and treated with GST or GST-SNCG (1 μmol/L) for Transwell assays. Alternatively, HCT116 cells were treated with the functional blocking anti-β1 specific antibody (5, 10, 20 μg/mL), integrin inhibitor RGD peptides (200 μmol/L), FAK inhibitor 14 (50 μmol/L), or MMP-2 inhibitor I (50 μmol/L) for 1 h, then GST or GST-SNCG (1 μmol/L) were added for migration or invasion assays as described previously [3].

### Screening of secreted proteins in conditioned medium (CM)

CM of HCT116 cells treated with GST or GST-SNCG (1 μmol/L) in the absence of FBS for 24 h was collected and measured using the Human Antibody L-series 507 Array (Cat# AAH-BLG-1-2, RayBiotech, Norcross, GA, USA) according to the recommended protocols. Briefly, all samples were biotinylated and added onto the blocked glass slide arrays pre-printed with capture antibodies. Streptavidin-conjugated fluorescent dye (Cy3 equivalent) was applied to the array. Final spot intensities were measured as the original intensities subtracting the background. Data were normalized to the positive controls in the individual slide.

### Gelatin zymography protease activity analysis of CM

CM of cancer cell lines treated with GST or GST-SNCG (1 μmol/L) was collected and filtered through 0.2 μm membrane filters and concentrated 20-50 times by centrifugation in Amicon Ultra-3 centrifugal filters (3 kDa, UFC500324, Millipore) at 4°C. Equivalent amounts of CM were applied to a 12% polyacrylamide gel containing 0.5 mg/mL gelatin. FBS (0.06 μL) was used as the positive control. After electrophoresis and washing twice with 2.5% Triton X-100, the gel was incubated in buffer containing 50 mM Tris-HCl, pH 7.4, 200 mM NaCl, and 10 mM CaCl_2_ at 37°C for 48 h, stained with Coomassie brilliant blue R250, and destained with 5% acetic acid containing 10% methanol.

### Statistical analysis

The correlation between SNCG and β1 integrin relative levels was determined by Spearman’s rank correlation analysis. Survival curves were estimated using the Kaplan–Meier method and compared by the log rank test. Hazard ratios (HRs) with 95% confidence intervals were estimated. Statistical comparisons were performed by un-paired two-tailed Student’s t test by SPSS standard version 19.0 (Chicago, USA). Acquired data were analyzed using Prism Software (GraphPad Prism). All data were expressed as mean ± SE. Differences of *P* < 0.05 or below were considered statistically significant and annotated on the figures accordingly.

## Results

### SNCG is identified as a peripheral membrane binding protein

Classically, SNCG is mainly detected in the cytoplasm with both free and vesicle-associated forms [3]. Our previous results demonstrated secretion of SNCG from tumor cells [3, 33]. Herein we confirmed this finding by comparing the levels of SNCG in the CM and lysates of several CRC cell lines (Fig.1a). Furthermore, we found that SNCG existed on cell membrane in CRC tissues (Fig. 1b) and exogenously added SNCG or GST-SNCG associated with cell membrane in different CRC cell lines (Fig. 1c), and the association in RKO cells could be detected as early as five minutes after treatment (Fig. 1d). These data support the notion that SNCG may change its intracellular localization and associate with subcellular structures in response to intracellular signaling or stress [35].

To understand the nature of SNCG binding to the cell membrane, we used Annexin A2, a known peripheral membrane binding protein [36], as the control. Cell membrane distribution of SNCG was similar to that of Annexin A2 (Fig. 1e). Moreover, endogenous SNCG in HT29 cells and exogenously added SNCG in LOVO cells had similar patterns of membrane distribution (Fig. 1f). Phase separation of membrane proteins revealed that SNCG was exclusively partitioned into the aqueous phase (Fig. 1g), indicating that SNCG is a peripheral membrane binding protein. These results also suggest that SNCG bound tumor cell membranes via specific membrane-associated entities.

### SNCG associates with β1 integrin and upregulates activated β1 integrin in CRC cells

**Fig. 2:**
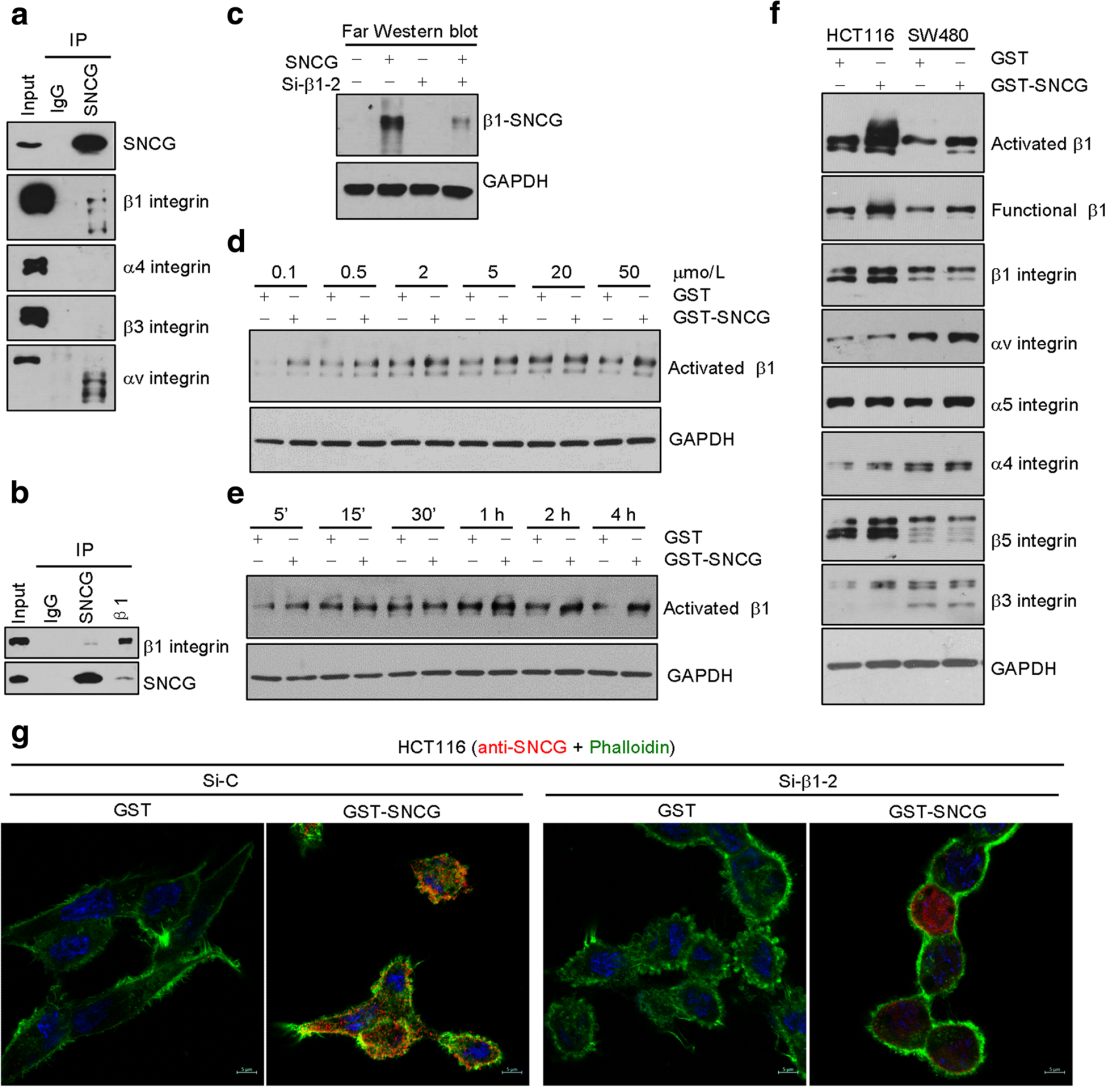
SNCG protein is associated with β1 integrin and activates β1 integrin. **a**-**b**. Coimmunoprecipitation. Cell membrane proteins of HCT116 cells were collected and subjected to immunoprecipitated (IP) using anti-SNCG (**a**), anti-SNCG or anti-β1 integrin antibody (**b**). The IP proteins or total cell lysates were analyzed by Western blot. Normal IgG served as the negative control. **c**. Far-Western blot analysis. HCT116 cells were transfected with control siRNA (lane 1-2), and specific siRNA-β1-2 (lanes 3-4) for 48 h. Cells were treated without (lane 1, 3) or with 1 μmol/L rhSNCG (lane 2, 4). Cell lysates were subjected to SDS-PAGE and transferred to NC membrane. β1 integrin (prey protein) on the membrane is detected with SNCG (bait protein). More SNCG was associated with membrane β1 integrin in SNCG-treated cells than that in the control cells (lane 1, 2). Correspondingly, less SNCG were detected in β1 integrin knock-down cells than that in control cells (lane 2, 4). **d**-**e**, Effect of concentration and time treatment of SNCG on activated β1 integrin. HCT116 cells were stimulated with GST or GST-SNCG at various concentrations for 60 min (**d**) or at fixed concentration (1 μmol/L) for various times (**e**). Cell lysates were analyzed with the HUTS-21 mAb recognizing the activated form of β1 integrin. **f**, GST-SNCG treatment (1 μmol/L) upregulated activated β1 integrin subunit in HCT116 and SW480 cells. **g**, Colocalization of SNCG with F-actin. HCT116 cells grown on coverslips were transiently transfected with control siRNA or β1-specific siRNA-2. After 72 h, cells were treated with GST or GST-SNCG (1 μmol/L) for 60 min. Cells were fixed and stained with anti-SNCG (red) and FITC-Phalloidin (green). Colocalization of SNCG and F-actin was shown in yellow. Nuclei were counterstained with DAPI (blue). Scale bars, 5 μm

To find the molecule(s) recruiting SNCG to cell membrane, we performed co-immunoprecipitation with anti-SNCG antibody to detect several known cell membrane proteins (i.e. EGFR, HER-2, integrins). Of all the tested proteins, only the β1 integrin was precipitated by anti-SNCG from HCT116 cell lysates (Fig. 2a). A reciprocal coimmunoprecipitation with anti-β1 integrin validated the endogenous SNCG-β1 integrin interaction (Fig. 2b). Immunoprecipitation assay may detect both direct and indirect associations, so the far Western blot assay is often used to confirm the direct interaction [37]. With this technique, β1 integrin (prey protein) on the membrane was probed by SNCG (bait protein) (Fig. 2c, lane 2). When the expression of β1 integrin was silenced by transfection with a specific siRNA (si-β1-2) for 72 h (Additional file 1: Figure S1A-C), less SNCG-β1 integrin complex was detected (Fig. 2c, lane 4), suggesting that SNCG was associated with β1 integrin. Next, we investigated the influence of SNCG on β1 integrin with the antibody HUTS-21, which specifically recognizes the active conformation of β1 integrin [38]. As shown in Fig. 2d and e, exogenously added GST-SNCG promoted levels of activated β1 integrin in a dose- and time-dependent manner. SNCG-promoted β1 integrin activation was confirmed in HCT116 and SW480 cells (Fig. 2f), however, no significant changes were observed in other integrin subunits detected (Fig. 2f). Since SNCG can directly participate in microtubule regulation [10] and β1 integrin binds to F-actin [39], we wonder whether the SNCG-integrin β1 interaction could lead to targeting of SNCG to F-actin cytoskeleton. We assessed the colocalization of SNCG with F-actin by confocal immunofluorescence staining. As shown in the Fig. 2g, SNCG colocalized with F-actin in HCT116 cells, which was mediated by β1 integrin, because knockdown of β1 integrin evidently decreased the colocalization (Fig. 2g).

### β1 integrin is required for SNCG-promoted tumor cell migration and invasion

**Fig. 3:**
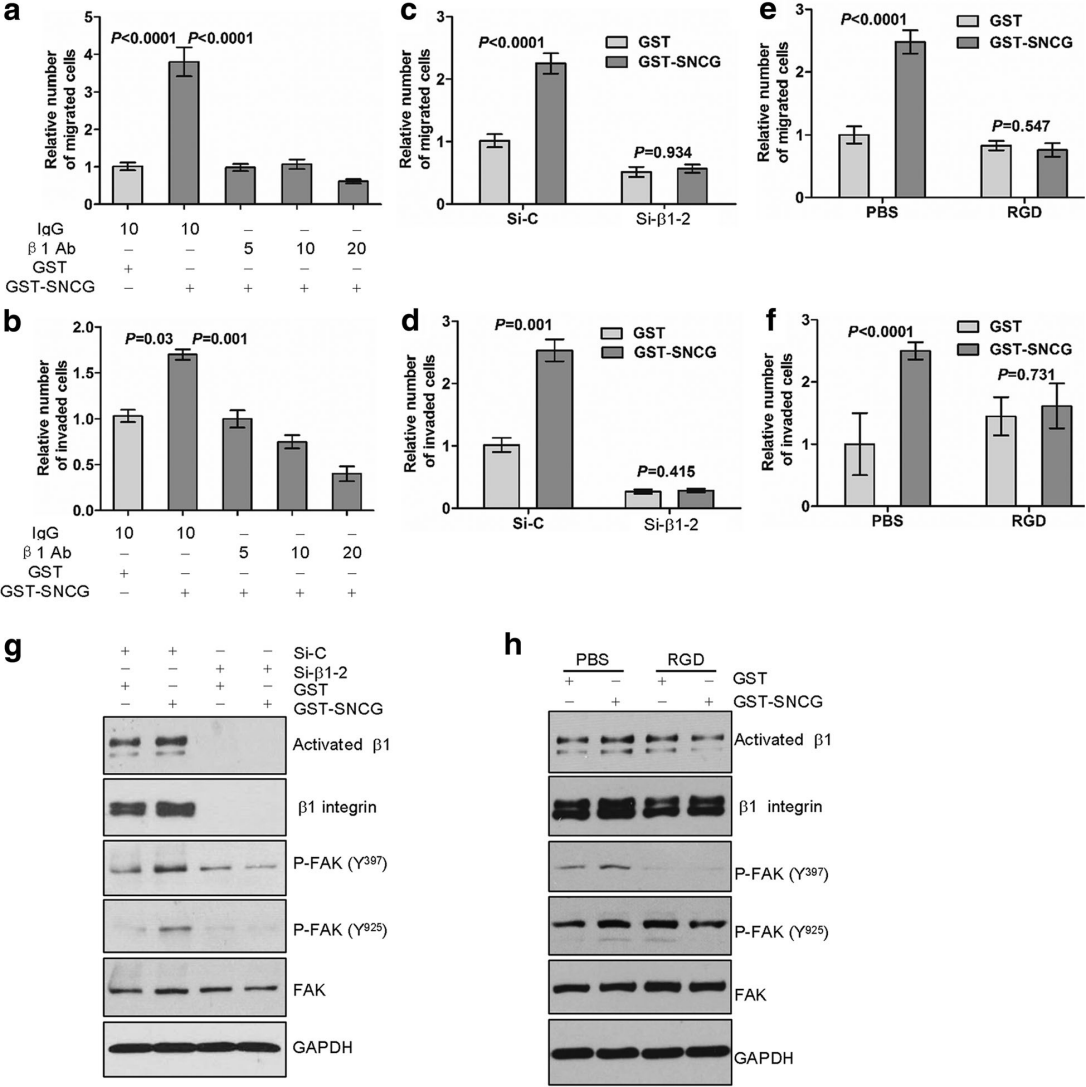
Integrin β1 is required for enhancement of SNCG on tumor cell migration and invasion. **a**-**b**, The functional blocking antibody for β1 integrin subunit (5, 10, 20 μg/mL) was added in the upper compartment of migration or invasion chambers stimulated with or without GST-SNCG (1 μmol/L) for 24 h for migration (**a**) or 48 h for invasion (**b**). **c**-**d**, HCT116 cells were transfected with control siRNA, and β1-specific siRNA-2 for 48 h. then cells were treated with or without GST-SNCG (1 μmol/L) for 24 h for migration (**c**) or 48 h for invasion (**d**). **e**-**f**, HCT116 cells were treated with PBS or 200 μmol/L RGD for 30 min and then treated with or without GST-SNCG (1 μmol/L) for 24 h for migration (**e**) or 48 h for invasion (**f**). Graphed data represent the mean ± SE from at least six 200-power field for each condition, two-sample t-test. **g**, HCT116 cells were transfected with control siRNA, and β1-specific siRNA-2 for 48 h. Then cells were treated with or without GST-SNCG (1 μmol/L) for 30 min and cell lysates were analyzed by Western blot. **h**, HCT116 cells were treated with PBS or 200 μmol/L RGD for 30 min. Then cells were treated with or without GST-SNCG (1 μmol/L) for 30 min and cell lysates were analyzed by Western blot

To evaluate whether β1 integrin is required for SNCG-promoted tumor cell motility, the function-blocking antibody against β1 integrin was added in the upper chambers with or without SNCG stimulation. We found that SNCG-promoted HCT116 cells migration (Fig. 3a) and invasion (Fig. 3b) was markedly suppressed by this antibody in a concentration-dependent manner, suggesting that β1 integrin contributes to SNCG-induced tumor cell motility. Moreover, silencing of β1 integrin expression in HCT116 cells by siRNA counteracted both SNCG-promoted migration (Fig. 3c) and invasion (Fig. 3d). Blockade of SNCG-promoted motility were also achieved by treating HCT116 cells with the integrin inhibitor RGD peptide (Fig. 3e, f). Similar inhibitory effects on SNCG-increased migration were further observed in SW480 cells by the function-blocking antibody or knockdown of β1 integrin (Additional file 1: Figure S2A-B).

To elucidate the mechanism underlying SNCG-promoted cell motility, we examined the changes in the activity of β1 integrin and FAK. SNCG enhanced activation of β1 integrin (Fig. 3g, h) and phospho-FAK (both Tyr^397^ and Tyr^925,^ Fig. 3g, h) levels were inhibited by β1 integrin knockdown (Fig. 3g) or RGD peptide (Fig. 3h) in HCT116 cells. Inhibition of SNCG-enhanced activation of p-FAK (Y^397^) and p-FAK (Y^925^) by silencing β1 integrin was additionally found in SW480 cells (Additional file 1: Figure S2C). These results suggested that SNCG could increase FAK phosphorylation through β1 integrin. Thus, SNCG-enhanced cell migration and invasion was β1 integrin-dependent and SNCG could activate β1 integrin-FAK signal pathway.

### The enhancement of migration and invasion mediated by SNCG is inhibited by abrogating FAK activation

**Fig. 4:**
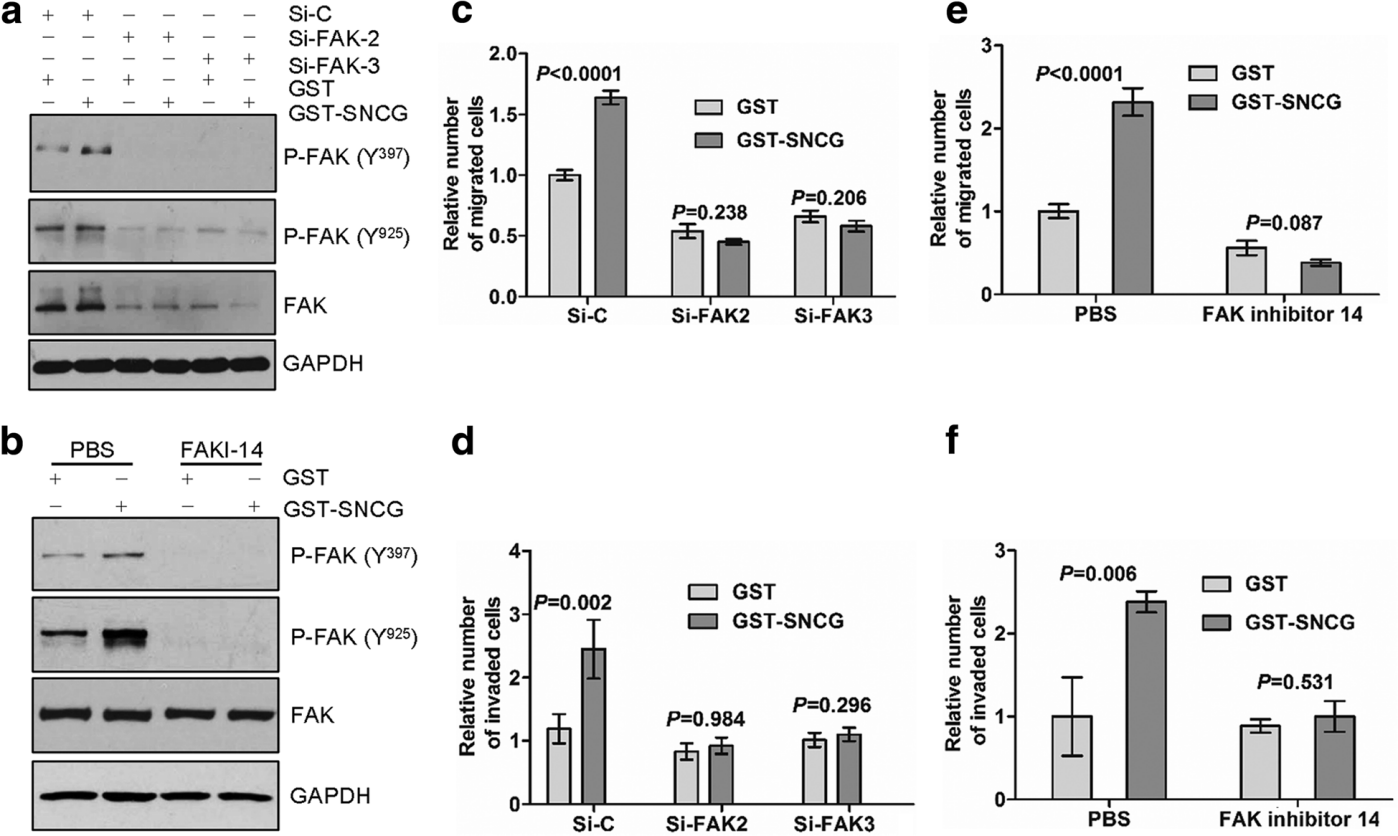
FAK is essential for SNCG-enhanced tumor cell migration and invasion. **a**-**b**, HCT116 cells were transfected with control siRNA and FAK-specific siRNA-2, -3 for 48 h. Then cells were treated with or without GST-SNCG (1 μmol/L) for migration (**a**) or invasion (**b**). **c**-**d**, HCT116 cells were treated with PBS, 50 μmol/L FAK inhibitor 14 for 30 min and then treated with or without GST-SNCG (1 μmol/L) for migration (**c**) or invasion (**d**). Graphed data represent the mean ± SE from at least six 200-power field for each condition, two-sample t-test.**e**-**f**, HCT116 cells were transfected with control siRNA (lane 1-2), and FAK-specific siRNA-2 (lane 3-4) and -3 (lane 5-6) for 72 h. cells were treated with or without GST-SNCG (1 μmol/L) for 30 min and cell lysates were analyzed for activated and total β1 integrin (**e**) or activated and total FAK (**f**)

Next, we assessed the effect of FAK on SNCG-promoted motility. HCT116 cells were transfected with FAK-specific siRNA (Additional file 1: Figure S2D, E) or treated with FAK inhibitor 14. Both treatments abolished SNCG-induced FAK phosphorylation (Fig. 4a, b). Consistently, SNCG-promoted migration (Fig. 4c, e) and invasion (Fig. 4d, f) were attenuated. These results indicated that FAK was involved in SNCG-promoted tumor cell migration and invasion. In contrast to SNCG-induced FAK phosphorylation, silencing β1 integrin or FAK altered neither ERK activation nor phospho-Src (Tyr^416^, Tyr^527^) levels in HCT116 or SW480 cells (Additional file 1: Figure S3A-C).

### As an indicator of adverse prognosis, SNCG level positively correlates with activated β1 integrin and p-FAK (Y^397^) in CRC tissues

**Fig. 5:**
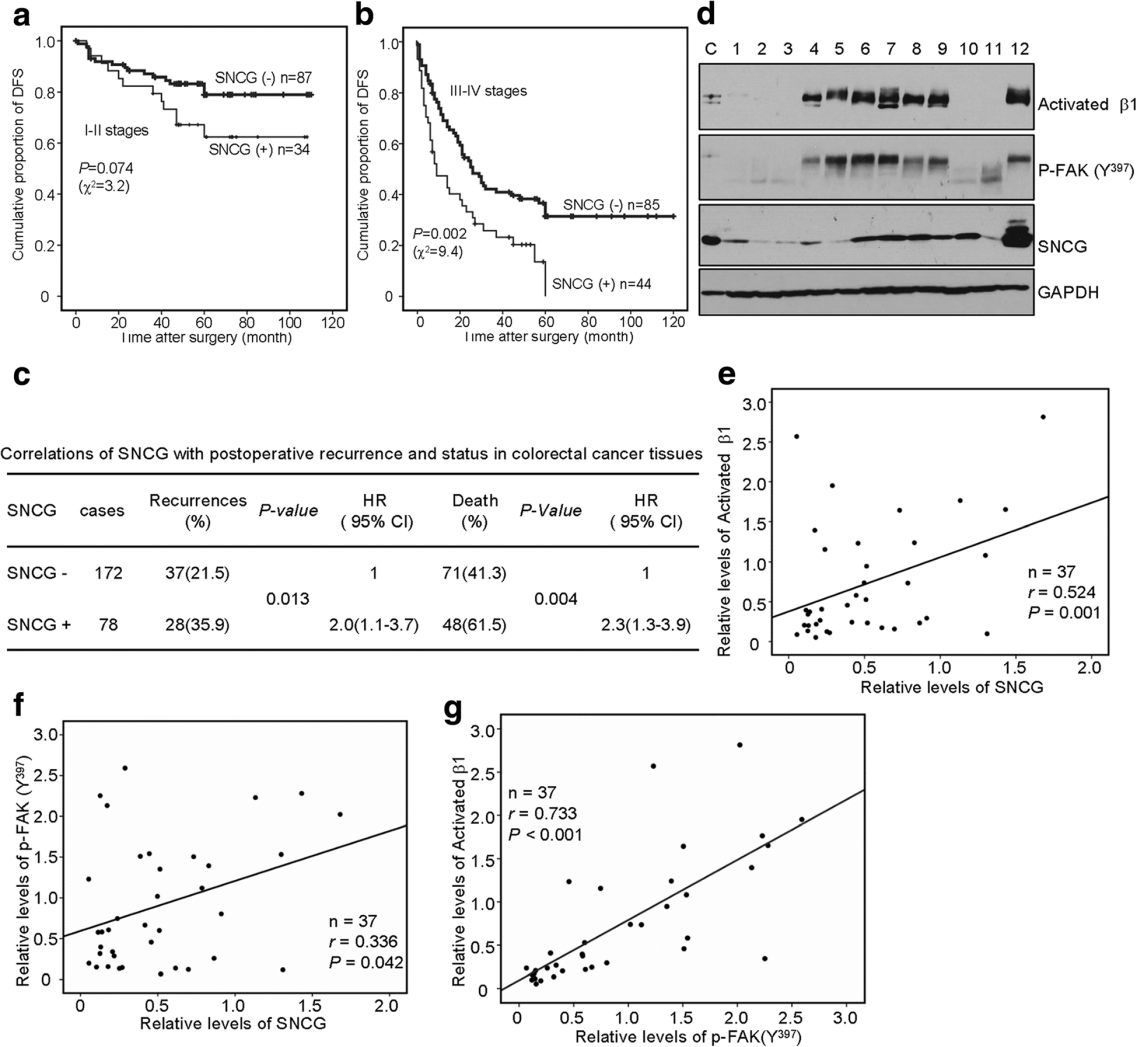
SNCG is an indicator of adverse prognosis and positively correlates with activated β1 integrin, p-FAK (Y^397^) in CRC tissues. **a**-**b**, Kaplan-Meier estimation of disease-free survival (DFS) for stage I-II (**a**) and III-IV (**b**) colorectal adenocarcinoma patients according to SNCG levels. **c**, Correlations of SNCG levels in CRC tissues with post-operative recurrence and status. **d**, Representative blots from three independent experiments were presented. Protein levels of SNCG, activated β1 integrin, and p-FAK (Y^397^) in clinical colon cancer tissue samples were evaluated by Western blot analysis. In order to increase the reproducibility, HCT116 cell lysates were used in each blot as the internal control (**c**) to minimize the effect of band intensity variation. GAPDH was used as the loading control. **e**-**g**, Correlation between the relative protein levels of activated β1 integrin levels and p-FAK (Y^397^) (**e**), SNCG and active β1 integrin (**f**), and SNCG and p-FAK (Y^397^) (**g**) were plotted as a scatter plots

SNCG levels were detected by immunohistochemistry in tissues from 250 patients with CRC. Higher expression of SNCG was associated with decreased disease-free survival (DFS) time of the patients with stage I-II (Fig. 5a, *P* = 0.074) or stage III-IV (Fig. 5b, *P*= 0.002) in Kaplan-Meier analysis. Moreover, stage III-IV patients with higher SNCG levels all fell into disease survival less than 5 years after operation (Fig. 5b). These data were supported by the finding that SNCG did not influence growth of tumor cells, but promoted tumor cell migration and invasion [3]. Furthermore, we found that high SNCG levels clearly correlated with recurrence (HR = 2.0 (1.1-3.7), *P* = 0.013) and poor prognosis (HR = 2.3 (1.3-3.9), *P* = 0.004) in this cohort of patients (Fig. 5c), which were consistent with our previous results 5, 6].

Based on the effect of SNCG on activated β1 integrin and p-FAK (Y^397^) in vitro, we investigated their associations in a cohort of frozen CRC tissues (n = 37) by Western blot analysis (Fig. 5d). After quantification, significantly positive correlations were observed between SNCG and activated β1 integrin (Fig. 5e, *r* = 0.524; *p* = 0.001), SNCG and p-FAK (Y^397^) (Fig. 5f, *r* = 0.336; *p* = 0.042), and activated β1 integrin and p-FAK (Y^397^) (Fig. 5g, *r* = 0.733; *p* = 0.0001), confirming the existence of SNCG-β1-FAK pathway in CRC tissues.

### Exogenously added SNCG remodels the microenvironment of tumor cells and increases MMP-2 activity through β1 integrin

**Fig. 6:**
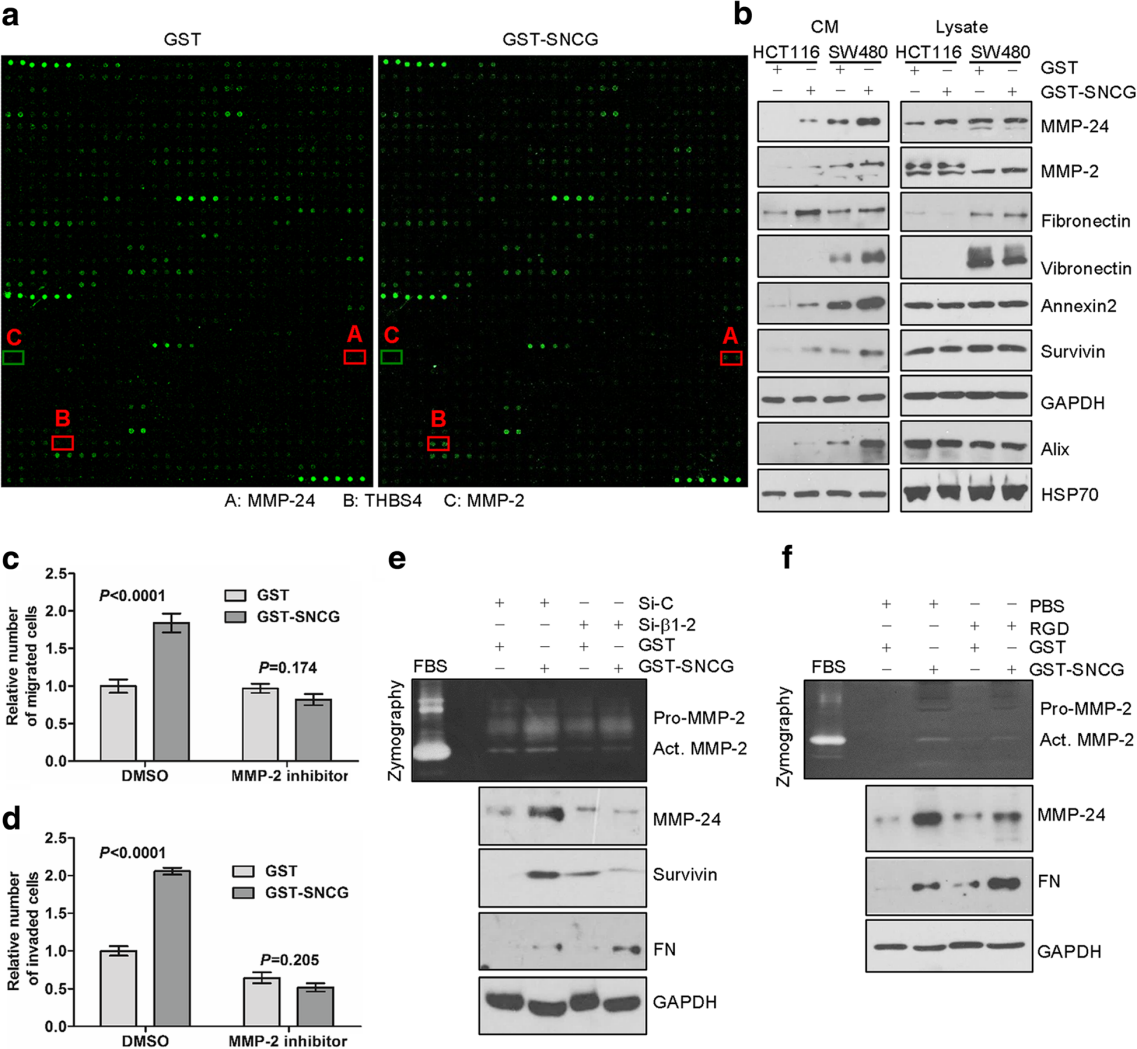
Exogenously added SNCG remodels the microenvironment of tumor cells and increases MMP-2 activity by β1 integrin. **a**, Antibody array screening of CM from GST and GST-SNCG-treated HCT116 cells. **b**, CM (left panel) and whole cell lysates (right panel) from HCT116 and SW480 cells treated with or without GST-SNCG (1 μmol/L) were subjected to Western blot analysis. Representative blots from three independent experiments were presented. **c**-**d**, HCT116 cells were treated with diluent, 50 μmol/L MMP-2 inhibitor for 40 min, then 1 μmol/L GST or GST-SNCG was added in the cell medium for migration (**c**) or invasion (**d**) assay. Migrated or invaded cells were quantitated after 24 h or 48 h, respectively. Error bars, SE of three determinations. **e**-**f**, Western blot and gelatin zymography analysis. CM from HCT116 cells treated with GST or GST-SNCG (1 μmol/L) in β1 integrin knock-down (**e**) or RGD-treated cells (**f**) was analyzed by gelatin zymography with FBS as the positive control, and secreted protein levels were analyzed by Western blot

Because SNCG promotes tumor cell invasion and positively correlated with distant metastasis [1–7], we investigate whether SNCG affects microenvironment of tumor cells. Relative expression of proteins in CM from HCT116 cells treated with GST or GST-SNCG was analyzed by the antibody array (Fig. 6a). Soluble membrane-type 5 matrix metalloproteinase (MT5-MMP/MMP-24) was identified as a significantly upregulated protein by SNCG treatment (fold change = 2.22), which was validated in the supernatants of CRC cell lines (Fig. 6b, Additional file 1: Figure S4A). This result was consistent with the finding that MT5-MMP tends to shed from cell surface as soluble proteases for extracellular matrix remodeling processes [40]. Although signal of THBS4 increased 2.6 fold times after treatment with GST-SNCG in HCT116 cells (Fig. 6a), we cannot validate this result by Western blot in SW480 or LOVO cells (Additional file 1: Figure S4A). No signal of MMP-2 was observed by antibody array screening (Fig. 6a), however we did notice elevated MMP-2 in CM of SNCG-stimulated HCT116 and SW480 cells (Fig. 6b). The difference may be due to different affinity of anti-MMP-2 antibody used in the assays. SNCG could promote secretion of MMP-2 as early as 5 min after SNCG treatment (Additional file 1: Figure S4B). Since MT5-MMP activates progelatinase A [40, 41] in tumor tissues and facilitates tumor progression [41], we then sought to explore whether SNCG-stimulated motility was mediated by activation of MMP-2. SNCG-enhanced migration (Fig. 6c) and invasion (Fig. 6d) were inhibited by treatment with a specific inhibitor of MMP-2 activity (MMP-2 inhibitor I). This indicates that proteolytic activity of MMP-2 could be responsible for SNCG-enhanced motility of HCT116 cells, at least in part. To confirm the correlation of SNCG with activated MMP-2, we performed gelatin zymography. SNCG increased proteolytic activity of MMP-2 in the CM of HCT116 cells compared with control, though, MMP-9 secretion was barely detectable in gelatin zymogram (Fig. 6e, f). Furthermore, SNCG-enhanced MMP-2 activity was inhibited by knockdown of β1 integrin (Fig. 6e) or treatment with RGD (Fig. 6f). The results were consistent with the idea that integrin-mediated signaling pathways are involved in regulation of MMP-2 expression and cell invasion in tumor cells [42].

In addition to MMPs, we observed that SNCG enhanced secretion of several proteins which were not included in the antibody array used, such as extracellular matrix fibronectin (FN), vitronectin (VN), and inhibitor of apoptosis survivin, exosome marker Alix (Fig. 6b, left panel), but intracellular abundance of these proteins were largely unaltered (Fig. 6b, right panel), indicating that either the secretion of these proteins or their extracellular stability was stimulated in SNCG-treated cells. SNCG-promoted secretion of MMP-24 and survivin was abolished by β1 integrin knockdown or RGD, however, secretion of FN was elevated (Fig. 6e, f), suggesting that SNCG may utilize multiple mechanisms to affect the profiles of secretome.

## Discussion

In the present study, our results indicated that SNCG was involved in β1 integrin/FAK signal pathway by interacting with β1 integrin and enhancing activation of β1 integrin and FAK, thereby promoting CRC cell migration and invasion. Integrins are regulated by conformational changes, clustering, and trafficking, and the regulating mechanisms differ dependent on integrins and cell types [43]. In circulating blood cells, integrins are activated by an inside-out-induced extended conformation that favors high-affinity ligand binding. In adherent cells, integrin activation occurs by an outside-in mechanism in the ECM with high concentration of available ligand, by avidity (integrin clustering), or by force [43]. Neither force nor high avidity seems to influence talin-induced IIbβ3 conformational change [44], suggesting that talin-regulated IIbβ3 activation may solely occur through conformational changes. β1 integrin inhibitor filamin can compete with talin for β1 cytoplasmic tail binding and inhibits β1 integrin activation [45]. Integrin trafficking plays an important role in the regulation of integrin turnover and integrin redistribution in adherent cells, especially during dynamic processes such as cell migration and invasion. In the present study, we demonstrated that extracellular SNCG bound β1 integrin on tumor cells and increased β1 integrin activation by using the antibody recognizing active conformation of β1 integrin, but the mechanism that triggers conformational changes from the bent to the extended conformation remains to be elucidated. It was found that SNCG regulated microtubule [10], we further disclosed the colocalization between SNCG with F-actin, which was diminished by knockdown of β1 integrin, indicating the existence of complex interactions among SNCG, β1 integrin, and cytoskeleton proteins. Our previous results showed that intracellular SNCG exhibits as both free and vesicle-associated forms [3] and SNCG has a dynamic localization and can associate with subcellular structures [35]. However, it remains unclear how SNCG bind β1 integrin. The binding site(s) of SNCG with β1 integrin interaction and how this binding affects β1 integrin conformation changes require further investigation in the following study.

As a key regulator, β1 integrin is involved in cancer progression [20, 21] and correlates with patient outcome [22]. Elevated activity of FAK has been described in a variety of human cancers [46]. Increased SNCG levels indicated poor outcome in colon cancer patients [5, 6]. Herein, we demonstrated that SNCG enhanced activation of β1 integrin and FAK in CRC cell lines, and SNCG level positively correlated with activated β1 integrin and phosphor-FAK (Y^397^) levels in colon cancer tissues. Altogether, our studies enlighten that the SNCG/β1/FAK signal pathway might be an important issue for the development of therapeutic strategies and diagnostic tools.

The cell microenvironment has a profound influence on the behavior, growth and survival of cells [47]. In the present study, we demonstrated that SNCG, as a secreted protein, also controlled secretion of many proteins in the tumor microenvironment, including ECM proteins such as fibronectin, vitronectin, the MMPs like MMP-2 and MMP-24. The data indicates that the balance between the ECM and membrane proteins is altered in the microenvironment of SNCG-treated cells, which was supported by that dysregulation or mutation of ECM components resulted in a broad range of pathological conditions [47]. However, many other proteins over-secreted by SNCG-treated cells are involved in cell migration and/or cell invasion and may also contribute to the role of SNCG in cancer metastasis. Fibronectin expression enhances tumor cell motility, cancer spread, and metastasis [48]. High levels of MMPs or aberrant MMPs expression is positively correlated with tumor progression, metastasis, and poor overall prognosis [29, 30, 49]. MT5-MMP may trigger tumor cell invasion or facilitate tumor progression by activating pro-gelatinase A on the tumor cell surface [40, 50]. Although inhibition of MMP-2 expression suppresses the invasiveness of tumor cells in several model systems [51, 52], the molecules inducing MMP-2 activation during tumor development had not been defined [53]. In the present study, increased MMP-24 shed and MMP-2 activation was observed in SNCG-treated tumor cells, suggesting that SNCG was an upstream regulator of MMP-24 and MMP-2. Howerer, MMP-9 activatity was barely detectable in gelatin zymogram in HCT116 cells treated with exogenous SNCG. MMP-9 was found to be upregulated in stable cell lines overexpressing SNCG [32] or downregulated in SNCG-knockdown cells [31]. Report of transient expression of SNCG in human breast cancer cells MDA-MB-435 [1] and a recent study of stable expression of SNCG in colon cancer cells LS 174T [54] did not alter the secretion of MMP2 or MMP9 in the CM. These discrepancies could be explained by the different cell types used or approaches of the role of SNCG played, such as our exogenously added SNCG in HCT116 cells, stably overexpressed SNCG in retinoblastoma Y79 cells [32] and in LS 174T [54] cells, or transiently overexpressed SNCG in MDA-MB-435 cells [1]. Nevertheless, our present study underscores SNCG as an important inducer of tumor cell motility, and the involvement of SNCG in remodeling extracellular microenvironment through β1-FAK and β1-MMP-2 signaling pathways. However, the mechanism of SNCG regulates protein secretion from tumor cells still awaits further characterization.

## Conclusions

In conclusion, extracellular SNCG binds β1 integrin on tumor cells and increases levels of activated β1 integrin, FAK, MMP-2, and protein secretion from tumor cells, which promotes β1-FAK signal pathway, remodels cell microenvironment matrix and subsequently affects tumor cell motility.

## Additional file

Additional file 1: **Table S1.** Detailed information of antibodies used in the study. **Table S2.** Clinicopathologic characteristics of patients (n=250). **Table S3.** Small interfering RNA sequences for β1 and FAK. **Figure S1.** Effect of small interfering RNA sequence on β1 protein expression. **Figure S2.** β1 integrin and FAK mediate SNCG-promoted tumor cell migration. **Figure S3.** Up-regulation of phospho-FAK induced by SNCG is blocked by β1 integrin knockdown, but has no effect on Src or Erk phosphorylation. **Figure S4.** Exogenously added SNCG promotes MMP-24 and MMP-2 secretion from colorectal cancer cells. (DOC 1280 kb)

## Abbreviations

CM: Conditioned medium
CRC: Colorectal cancer
ECM: Extracellular matrix
FAK: focal adhesion kinase
FAKI: FAK inhibitor 14
FN: fibronectin
HR: Hazard ratio
MMP: Matrix metalloproteinase
PMSF: Phenylmethanesulfomyl fluoride
RGD: Arg-Gly-Asp peptides
SDS-PAGE: Sodium Dodecyl Sulfate–Polyacrylamide Gel Electrophoresis
SiRNA: Small interfering RNA
SNCG: gamma-synuclein
VN: Vitronectin
