# Supplementary material for: Extracellular gamma-synuclein promotes tumor cell motility by activating β1 integrin-focal adhesion kinase signaling pathway and increasing matrix metalloproteinase-24, -2 protein secretion
Source: J Exp Clin Cancer Res. 2018 Jun 15;37:117. doi: 10.1186/s13046-018-0783-6 (PMC6003176; doi:10.1186/s13046-018-0783-6)
Supplement: Supplementary file 1 — Table S1. Detailed information of antibodies used in the study. Table S2. Clinicopathologic characteristics of patients (n=250). Table S3. Small interfering RNA sequences for β1 and FAK. Figure S1. Effect of small interfering RNA sequence on β1 protein expression. Figure S2. β1 integrin and FAK mediate SNCG-promoted tumor cell migration. Figure S3. Up-regulation of phospho-FAK induced by SNCG is blocked by β1 integrin knockdown, but has no effect on Src or Erk phosphorylation. Figure S4. Exogenously added SNCG promotes MMP-24 and MMP-2 secretion from colorectal cancer cells. (DOC 1280 kb) [file 13046_2018_783_MOESM1_ESM.doc]

**Additional file**

**Table S1. Detailed information of antibodies used in the study**

| **Antibody** | **company** | **Applications in the paper** | **Catalogue No.** | **clone** | **Source** |
| --- | --- | --- | --- | --- | --- |
| β1 integrin (Active conformation) | BD pharmingen | WB: 1: 100000 | 556048 | HUTS-21 | Mouse IgG2a |
| β1 integrin (Function-  Blocking) | Santa Cruz | WB: 1: 2000  Blocking: 10 μg/mL | Sc-13590 | P5D2 | Mouse, IgG1 |
| β1 integrin  (Total) | abcam | WB: 1: 500 | Ab134179 | EPR1040Y | Rabbit IgG |
| αv integrin | Santa Cruz | WB: 1: 500 | Sc-6617-R | Q-20-R | Rabbit, polyclonal |
| α4, α5, 3, 5 integrin | CST | WB: 1: 100 | #4749 | Integrin Antibody Sampler Kit | Rabbit, IgG |
| p-FAK (Y925) | CST | WB: 1: 1000 | #3284 |  | Rabbit, polyclonal |
| p-FAK (Y397) | CST | WB: 1: 500 | #8556 | D20B1 | Rabbit, IgG |
| FAK | CST | WB: 1: 2000 | #3284 | D2R2E | Rabbit, IgG |
| MMP-2 | CST | WB: 1: 1000 | #13132 | D8N9Y | Rabbit, IgG |
| Fibronectin | Santa Cruz | WB: 1: 500 | Sc-8422 | EP5 | Mouse, IgG1 |
| Vitronectin | Santa Cruz | WB: 1: 2000 | Sc-74485 | B-1 | Mouse, IgG1 |
| MT5-MMP | Abcam | WB: 1: 1000 | Ab39695 |  | Rabbit, polyclonal |
| THBS4 | Abcam | WB: 1: 2000 | Ab156258 |  | Rabbit, polyclonal |
| Annexin A2 | Novus | WB: 1: 1000 | H00000302-M02 | 1G7 | Mouse, IgG1 |
| Alix | CST | WB: 1: 500 | #2171 | 3A9 | Mouse, IgG1 |
| HSP70 | EPITMICS | WB: 1: 2000 | #1776-1 | EP1007Y | Rabbit, IgG |
| GAPDH | Proteintech | WB: 1: 200000 | 60004-1-Ig | 1E6D9 | Mouse, IgG2b |

WB: Western blot.

**Table S2. Clinicopathologic characteristics of patients (n=250)**

| **Cases** | **Gender** | | **Age (years)** | | **Tumor Size (cm) #** | | **Clinical stages** | | **Status** | |
| --- | --- | --- | --- | --- | --- | --- | --- | --- | --- | --- |
|  | Male | Female | <60 | ≥60 | <4 | ≥4 | I-II | III-IV | live | death |
| **250** | 132 | 118 | 101 | 149 | 94 | 154 | 121 | 129 | 131 | 119 |

# Two cases in tumor size were unknown

**Table S3. Small interfering RNA sequences for β1 and FAK**

| **Genes** | **Forward sequence**  **(5’3’)** | **Reverse sequence**  **(5’3’)** |
| --- | --- | --- |
| β1 integrin-1 | CCACAGCAGUUGGUUUUGCTT | GCAAAACCAACUGCUGUGGTT |
| β1 integrin-2 | GCACCAGCCCAUUUAGCUATT | UAGCUAAAUGGGCUGGUGCTT |
| β1 integrin-3 | GCAGCACAGAUGAAGUUAATT | UUAACUUCAUCUGUGCUGCTT |
| FAK-1 | GGGCAUCAUUCAGAAGAUATT | UAUCUUCUGAAUGAUGCCCTT |
| FAK-2 | CAGGUGAAGAGCGAUUAUATT | UAUAAUCGCUCUUCACCUGTT |
| FAK-3 | GAACCUCGCAGUCAUUUAUTT | AUAAAUGACUGCGAGGUUCTT |
| Negative control | UUCUCCGAACGUGUCACGUTT | ACGUGACACGUUCGGAGAATT |
| GAPDH | UGACCUCAACUACAUGGUUTT | AACCAUGUAGUUGAGGUCATT |

**Supplementary Figures**

**
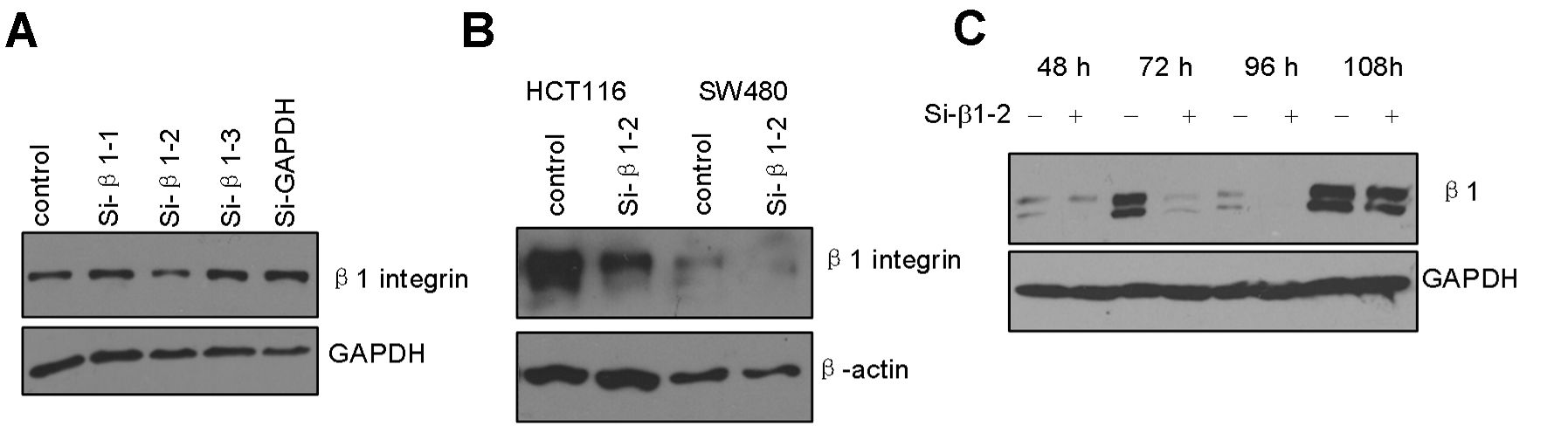
**

**Figure S1. Effect of small interfering RNA sequence on β1 protein expression.** β1 integrin-specific small interfering RNA (siRNA) and control siRNA sequence were transiently transferred into HCT116 (A-C) or SW480 (B) cells for 24 h (A, B) or 48-108 h (C). Lysates were subjected to Western blot.

**
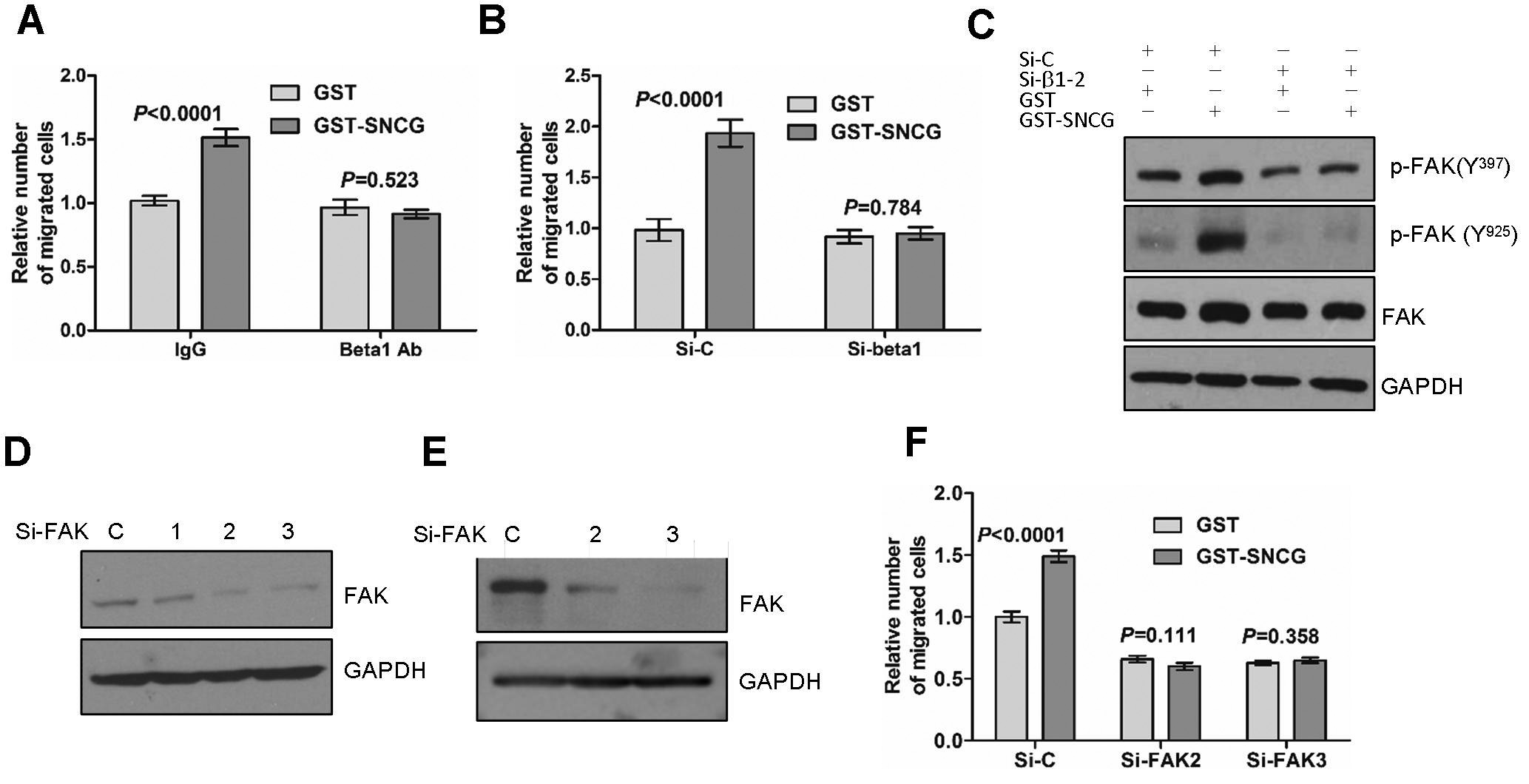
**

**Figure S2. β1 integrin and FAK mediate SNCG-promoted tumor cell migration.**

**A-B**, Migration. SW480 cells were treated with 20 μg/mL of the function blocking antibody agninst β1 integrin for 1 h (A) or transfected with β1-specific siRNA-2 (B) for 48 h, then cells were treated with GST or GST-SNCG (1 μmol/L) and performed migration assay. Graphed data represent the mean ± SE from at least six 200-power field for each condition, two-sample t-test. **C**, SW480 cells were transfected and treated as in (B), and cell lysates were analyzed by Western blot with indicated antibodies. **D-F**, control and FAK-specific siRNAs were transfected into HCT116 (D) and SW480 (E) cells for 48 h. Cell lysates were subjected to Western blot. **F**, SW480 cells were transfected with FAK-specific siRNA-2, -3 for 48 h, then cells were treated with GST or GST-SNCG (1 μmol/L) and performed migration assay. Data represent the mean ± SE from six 200-power field for each condition, two-sample t-test.

**
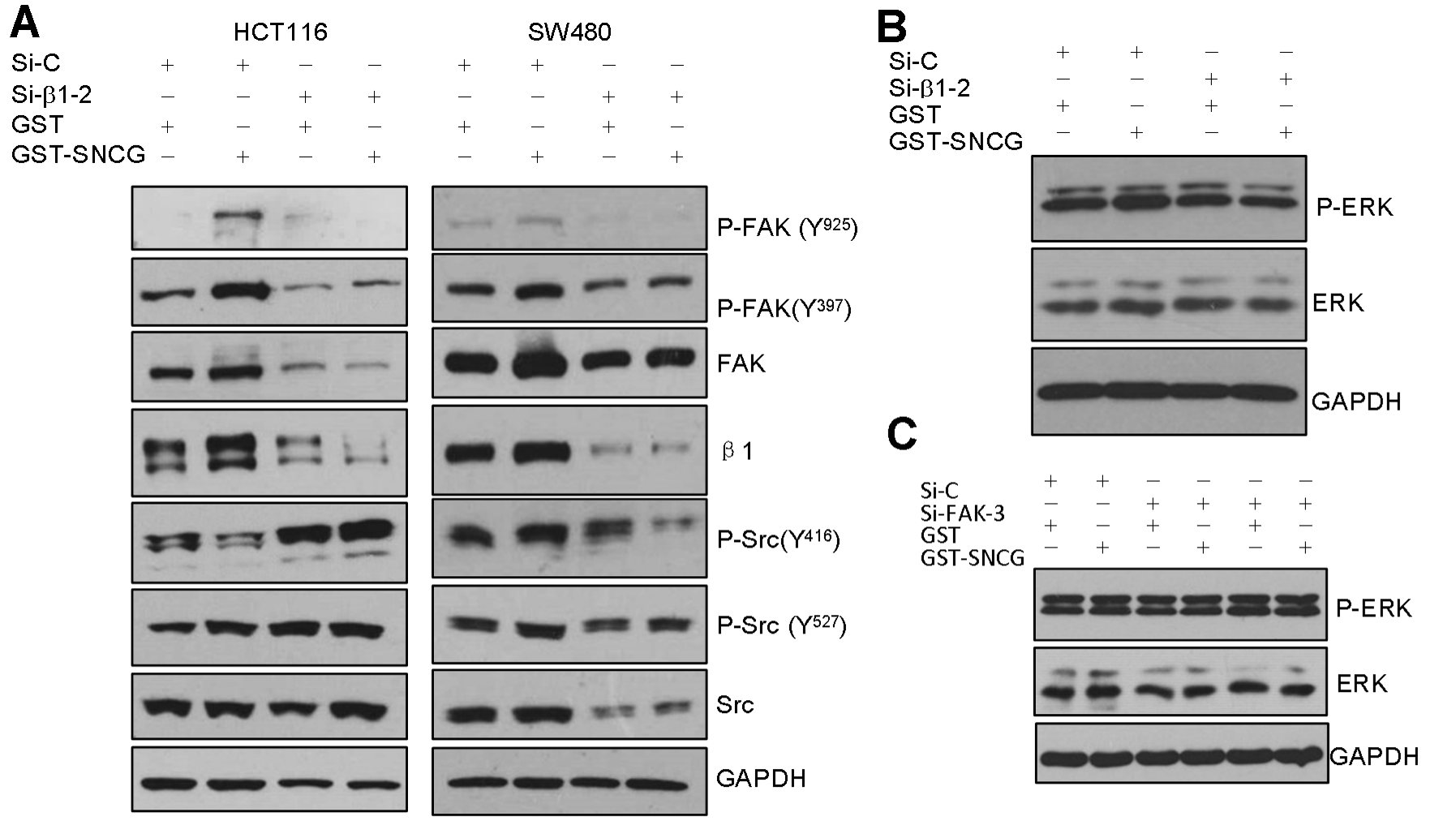
**

**Figure S3. Up-regulation of phospho-FAK induced by SNCG is blocked by β1 integrin knockdown, but has no effect on Src or Erk phosphorylation.**

**A**, HCT116 and SW480 cells were transfected with control and β1-specific siRNA-2 for 48 h, then cells were treated with or without GST-SNCG (1 μmol/L) for 30 min and cell lysates were analyzed for phosphorylated and total proteins. **B-C**, HCT116 cells were transfected with control siRNA, β1-specific siRNA-2 (B), or FAK-specific siRNA-2, -3 (C) for 48 h, then cells were treated with or without GST-SNCG (1 μmol/L) for 30 min. Cell lysates were analyzed for phosphorylated and total ERK, Src by Western blot.

**
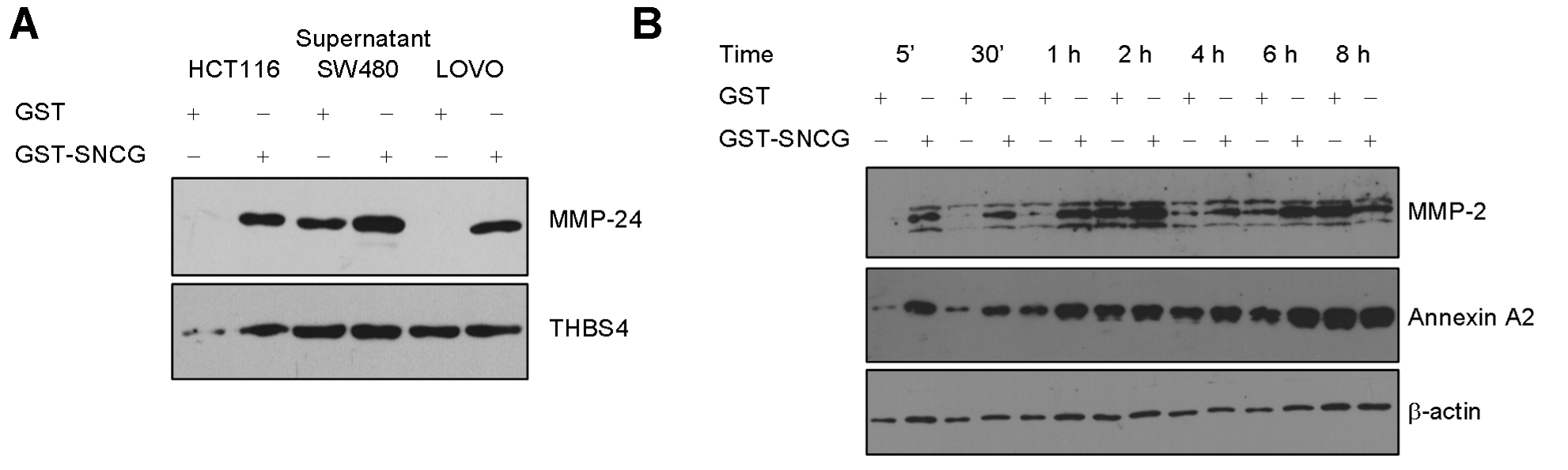
**

**Figure S4. Exogenously added SNCG promotes MMP-24 and MMP-2 secretion from colorectal cancer cells**.

**A**, Validation of microarray result by Western blot analysis. Conditioned media (CM) from HCT116, SW480, and LOVO cells treated with GST or GST-SNCG (1 μmol/L) for 24 h were subjected to Western blot analysis of MMP-24 and THBS4. **B**, Detection of MMP-2 secretion by Western blot. CM from HCT116 cells treated with GST or GST-SNCG (1 μmol/L) at various time points was harvested, concentrated, and 20 μg protein were subjected to Western blot analysis for MMP-2 secretion.
